# Effect of oxidative stress on sympathetic and renal vascular responses to ischemic exercise

**DOI:** 10.1002/phy2.47

**Published:** 2013-08-22

**Authors:** Matthew D Muller, Rachel C Drew, Jian Cui, Cheryl A Blaha, Jessica L Mast, Lawrence I Sinoway

**Affiliations:** Penn State Hershey Heart and Vascular Institute, Pennsylvania State University College of Medicine500 University Drive, Hershey, Pennsylvania, 17033

**Keywords:** Antioxidant, forearm blood flow, hyperoxia, muscle afferents, sympathetic nervous system

## Abstract

Reactive oxygen species (ROS), produced acutely during skeletal muscle contraction, are known to stimulate group IV muscle afferents and accentuate the exercise pressor reflex (EPR) in rodents. The effect of ROS on the EPR in humans is unknown. We conducted a series of studies using ischemic fatiguing rhythmic handgrip (IFRHG) to acutely increase ROS within skeletal muscle, ascorbic acid infusion to scavenge free radicals, and hyperoxia inhalation to further increase ROS production. We hypothesized that ascorbic acid would attenuate the EPR and that hyperoxia would accentuate the EPR. Ten young healthy subjects participated in two or three experimental trials on separate days. Beat-by-beat measurements of heart rate (HR), mean arterial pressure (MAP), muscle sympathetic nerve activity (MSNA), and renal vascular resistance index (RVRI) were measured and compared between treatments (saline and ascorbic acid; room air and hyperoxia). At fatigue, the reflex increases in MAP (31 ± 3 vs. 29 ± 2 mm Hg), HR (19 ± 3 vs. 20 ± 3 bpm), MSNA burst rate (21 ± 4 vs. 23 ± 4 burst/min), and RVRI (39 ± 12 vs. 44 ± 13%) were not different between saline and ascorbic acid. Relative to room air, hyperoxia did not augment the reflex increases in MAP, HR, MSNA, or RVRI in response to exercise. Muscle metaboreflex activation and time/volume control experiments similarly showed no treatment effects. While contrary to our initial hypotheses, these findings suggest that ROS do not play a significant role in the normal reflex adjustments to ischemic exercise in young healthy humans.

## Introduction

Both isometric and isotonic (rhythmic) exercises activate the sympathetic nervous system (Mark et al. 1985; Victor and Seals 1989; Victor et al. 1995). This activation is mediated by central command and feedback from the contracting skeletal muscle, termed the exercise pressor reflex (EPR). Thinly myelinated group III afferents (i.e., mechanoreceptors) respond primarily to mechanical stimuli while unmyelinated group IV afferents (i.e., metaboreceptors) primarily respond to metabolites of muscle contraction such as lactate, H^+^, K^+^, adenosine, and arachidonic acid (McCloskey and Mitchell 1972; Kaufman et al. 1983). In rats, reactive oxygen species (ROS) stimulate group IV muscle afferents (Delliaux et al. 2009) and exert an excitatory role on the EPR (Wang et al. 2009) but the effect of ROS on the EPR in healthy humans is unknown.

Oxidative stress is involved in the pathogenesis of several cardiovascular diseases (Harrison et al. 2003) and is defined as an imbalance between ROS production and the body's antioxidant defense systems. ROS production increases acutely during rhythmic muscle contraction (O'Neill et al. 1996; Reid and Durham 2002; Karamouzis et al. 2004; Rietjens et al. 2007). Using an exercise mode that prevents blood from entering or exiting the working muscle, it would be possible to experimentally isolate the effect of ROS on the EPR without confounding changes in oxygen delivery (i.e., blood flow). To this end, our laboratory (Sinoway et al. 1992; McClain et al. 1993; Ettinger et al. 1996; Mostoufi-Moab et al. 1998; Cornett et al. 2000) and others (Victor et al. 1987) have previously used ischemic fatiguing rhythmic handgrip (IFRHG) to investigate sympathetic neural mechanisms involved in the EPR independent of blood flow. Moreover, we recently demonstrated that the EPR (low intensity rhythmic plantar flexion) was augmented in patients with peripheral arterial disease (PAD) via an oxidative stress mechanism (Muller et al. 2012). Considering that resting limb ischemia and oxidative stress are hallmarks of PAD (Harrison et al. 2003), a better understanding of how the body responds to ischemic exercise could be clinically valuable.

Human studies have demonstrated that muscle sympathetic nerve activity (MSNA) and renal vascular resistance index (RVRI) both increase during handgrip exercise (Middlekauff et al. 1997; Momen et al. 2003; Kuipers et al. 2009). This sympathetically mediated vasoconstriction to inactive tissue, along with a moderate increase in heart rate (HR), effectively increases mean arterial pressure (MAP) in an effort to improve perfusion to the working forearm muscle. Considering this frame of reference, the primary aim of this study was to determine the effect of oxidative stress on the EPR in healthy young humans. We used IFRHG (to acutely increase ROS within skeletal muscle), ascorbic acid infusion (to scavenge free radicals), and hyperoxia inhalation prior to and during exercise (to further increase ROS production). By preventing blood flow into the working forearm muscle, we sought to isolate the reflex changes in HR, MAP, MSNA, and RVRI that occur when ROS stimulate muscle afferents (i.e., independent of oxygen delivery). We hypothesized that ascorbic acid would attenuate the EPR and hyperoxia would accentuate the EPR.

## Methods

### Subjects and design

This study used a repeated measures, crossover design and the primary independent variables were infusion (saline vs. ascorbic acid), breathing (room air vs. hyperoxia), and time (rest vs. exercise). All study protocols were approved in advance by the Institutional Review Board of the Penn State Milton S. Hershey Medical Center and conformed to the Declaration of Helsinki. Ten young (28 ± 2 years, six men, four women) subjects volunteered to participate and provided written informed consent. All subjects were normotensive, nonasthmatic, nonsmokers, not taking any prescription or vasoactive medication, and were in good health as determined by history and physical examination. They had an average height of 1.73 ± 0.04 m, weight of 74.6 ± 4.2 kg, and body mass index of 24.7 ± 1.0 kg/m^2^. Subjects refrained from caffeine, alcohol, and exercise for 24 h before the study and arrived to the laboratory following an overnight fast. All experiments were conducted in the morning hours in a dimly lit thermoneutral laboratory (22–25°C).

### Instrumentation

For all experiments, participants were supine and were outfitted with a 3-lead Electrocardiography (ECG) (Cardiocap/5, GE Healthcare, Waukesha, WI) to monitor HR, a finger cuff to monitor beat-by-by-beat blood pressure (BP) (Finometer, FMS, Arnhem, The Netherlands), and a pneumotrace to monitor respiratory movement. MSNA was measured by microneurography and renal blood flow velocity (RBV) was measured using transabdominal Doppler ultrasound, both as described below. During Visit 3, subjects also wore a nonrebreathing oronasal mask connected in series to a reservoir bag containing the hyperoxic (100% oxygen) gas mixture. For these experiments, arterial oxygen saturation (oximetry of the earlobe), end tidal CO_2_, and minute ventilation were monitored continuously (Respiratory Gas Monitor, Ohmeda 5250). All parameters were sampled at 200 Hz by a data acquisition system (PowerLab, ADInstruments, New Castle, Australia). Verbal and written time stamps were used to match hemodynamic and renal parameters during off-line analysis. Prior to each trial, an automated sphygmomanometer (Philips SureSigns Vs3, Andover, MA) was used to determine resting brachial artery blood pressure (in triplicate). These values were used to verify the Finometer cuff pressures.

Multifiber recordings of MSNA were obtained with a tungsten microelectrode inserted in the peroneal nerve of a leg. A reference electrode was placed subcutaneously 2–3 cm from the recording electrode. The recording electrode was adjusted until a site was found in which muscle sympathetic bursts were clearly identified using previously established criteria (Vallbo et al. 1979). The nerve signal was amplified, band-pass filtered with a bandwidth of 500–5,000 Hz, and integrated with a time constant of 0.1 sec (Iowa Bioengineering, Iowa City, IA). The nerve signal was also routed to a loudspeaker and a computer for monitoring throughout the study.

Doppler ultrasound (HDI 5000, ATL Ultrasound, Bothell, WA) was used to measure RBV. The artery was scanned with a curved array C5-2 transducer using a transabdominal approach, as previously described (Momen et al. 2003; Sauder et al. 2008). Renal artery diameter was not measured due to the fact that low frequency transducers (needed to measure a relatively deep artery) do not have high spatial resolution. Renal vascular resistance index (RVRI) was calculated as MAP/RBV and expressed as a percent change (%Δ) from baseline, which normalizes for interindividual differences in Doppler angle and depth. An increase in RVRI indicates renal vasoconstriction (Conboy et al. 2010; Momen et al. 2005, 2003; Muller et al. 2013; Patel et al. 2013; Sauder et al. 2008)

### Ischemic exercise protocols

**Figure 1:**
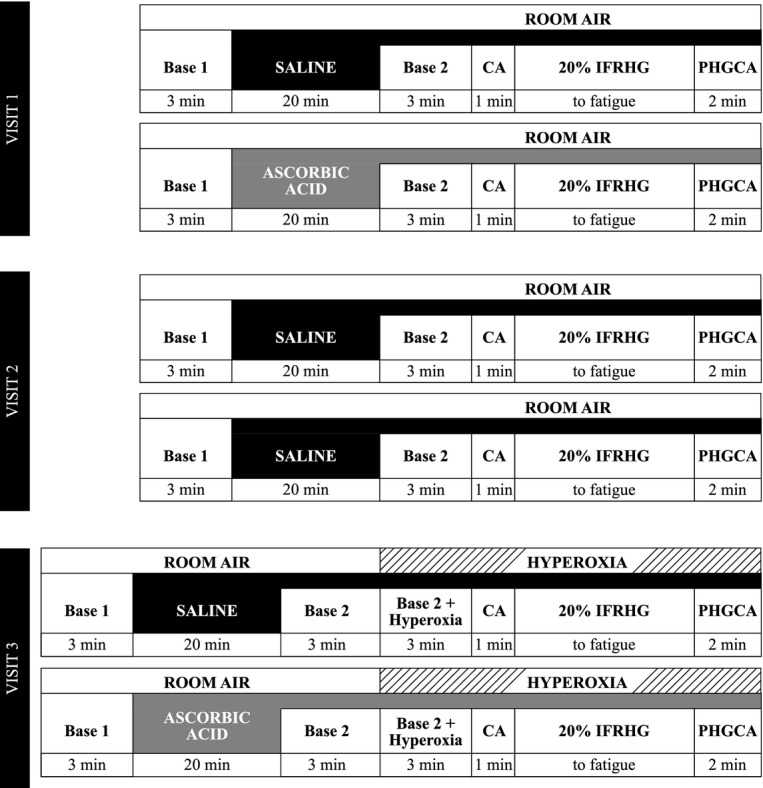
Experimental timeline. Please see text for details. CA, circulatory arrest via inflation of arm cuff to suprasystolic pressure; IFRHG, ischemic fatiguing rhythmic handgrip; PHGCA, posthandgrip circulatory arrest.

Subjects reported to the laboratory for two or three total visits separated by 2 to 4 weeks. The protocol for each visit is depicted in Figure 1 and included two separate bouts of IFRHG at 20% maximal voluntary contraction (MVC) following infusion of either saline or ascorbic acid. During Visit 1 (*n* = 10), subjects received intravenous infusions of saline and then ascorbic acid (opposite arm used for handgrip). During Visit 2 (*n* = 6), subjects received two separate infusions of saline as a time/volume control. During Visit 3 (*n* = 9), subjects received saline and then ascorbic acid and also breathed 100% oxygen prior to and during exercise. Visits occurred chronologically (i.e., Visit 1 was always first and Visit 3 was always last).

Saline was given as a loading dose (100 mL in 20 min) followed by a maintenance dose (20–30 mL within 60 min). Ascorbic acid was given as a loading dose (45 mg/kg in 100 mL saline) over 20 min followed by a maintenance dose (15 mg/kg in 33 mL saline) for the remainder of the study. Due to the high concentration given (i.e., sufficient to scavenge ROS), ascorbic acid was always given second. Following the first exercise bout, a 20–30 min rest period occurred prior to starting the second infusion.

Upon arrival at the laboratory, MVC was determined in triplicate with a dynamometer (Stoelting Company, Wood Dale, IL) and 20% of this value was calculated for use during the trial. Participants were familiarized to the Borg rating of perceived exertion scale (where 6 = very, very light and 20 = maximal exertion) (Borg 1982) and it was emphasized that the upcoming IFRHG trial should continue until the hand and forearm reached a score of “20”. To make the forearm ischemic prior to exercise, a 1-min period of circulatory arrest (CA) was achieved by inflating an upper arm cuff to suprasystolic pressure. This cuff remained inflated for the duration of IFRHG (30 contractions/min at 20% MVC until fatigue) and for an additional 2-min phase of posthandgrip circulatory arrest (PHGCA) to isolate the muscle metaboreflex. During Visit 3, a 3-min period of hyperoxic breathing occurred prior to CA (i.e., to allow ROS to enter the forearm) and continued until the completion of 2 min of PHGCA (Fig. 1). Based on prior publications (Crawford et al. 1997; Mak et al. 2002) and our follow-up study (details below), 3 min of hyperoxia raises arterial oxygen saturation and elicits forearm vasoconstriction (∼40% reduction from room air baseline). To provide visual feedback to the subject during exercise, the handgrip dynamometer was interfaced to a custom device using an analog meter display. Care was taken to avoid the Valsalva maneuver during IFRHG and researchers ensured that the subject did not contract the biceps or shoulder during forearm muscle contraction.

### Follow-up study

Based on the findings from the ischemic exercise studies (Visits 1–3), additional trials were conducted in a separate group of subjects (*n* = 8, four men and four women). These trials served three distinct purposes: (1) to determine the time course of hyperoxia-mediated forearm vasoconstriction (i.e., to confirm hyperoxia inhalation for 3 to 5 min elicited physiological effects in the freely perfused forearm); (2) to evaluate the effect of systemic ascorbic acid on forearm blood flow (FBF) before and during 100% hyperoxia; and (3) to document plasma levels of ascorbic acid before and after breathing 100% hyperoxia. These follow-up studies could not be performed during the ischemic exercise experiments because an occlusion cuff covered the brachial artery.

Following the placement of two intravenous catheters (one for infusion and one for blood samples) and baseline measurements, subjects underwent 5 min of breathing 100% hyperoxia (same as Visit 3). Subjects were unaware the time at which they were switched from room air to hyperoxia. After a 20-min recovery period, ascorbic acid infusion occurred (same dose as above) and then a second 5-min bout of 100% hyperoxia breathing occurred. Venous blood samples were obtained before and after each bout of hyperoxia (four samples total) to determine ascorbic acid levels. The rationale for these additional studies was based on prior work from our laboratory (Crawford et al. 1997; McNulty et al. 2005, 2007; Gao et al. 2012) and others (Narkowicz et al. 1993; Milone et al. 1999; Mak et al. 2002). Along with BP (Finometer), HR (ECG), and respiratory parameters (respiratory gas monitor, Ohmeda 5250), FBF was measured by duplex ultrasound (HDI 5000, ATL). Briefly, a 5 to 12 MHz linear transducer was placed over the brachial artery and the insonation angle was less than 60°. Brachial artery mean blood flow velocity was acquired in pulsed Doppler mode and velocity waveforms were synchronized to the PowerLab data acquisition system by a Doppler audio transformer (Herr et al. 2010). Brachial artery diameter measurements were obtained at end diastole during the last 15 sec of each minute. FBF was calculated by multiplying the cross-sectional area (πr^2^) of the vessel by mean blood flow velocity and by 60. Forearm vascular conductance (FVC) was calculated as FBF/MAP and expressed as a percent change from baseline, consistent with prior reports (Wilson et al. 2007). Ascorbic acid levels were measured by Quest Diagnostic Nichols Institute (San Juan Capistrano, CA).

### Statistical analysis

All statistical analyses were conducted using IBM SPPS 19.0, and graphics were produced using Microsoft Excel and Adobe Illustrator CS5. Normality was confirmed by the Kolmogorov–Smirnov test (i.e., *P* > 0.05 for all physiological measurements). Separate two-way repeated measures analyses of variance (time and infusion; time and breathing) were conducted to address each hypothesis. To more simply characterize the EPR (i.e., peak responses), the changes (Δ) in HR, MAP, MSNA, RBV, and RVRI relative to the postinfusion baseline (Base 2) were calculated by using data from the last 30 seconds of IFRHG. Changes from baseline during PHGCA were also calculated to assess the muscle metaboreflex, consistent with previous handgrip experiments (Kuipers et al. 2009; Delaney et al. 2010). These changes from baseline were analyzed with paired samples t-tests. For the follow-up study, a 2 trial (pre- and postascorbic acid infusion) by 6 (base, 1 min, 2 min, 3 min, 4 min, 5 min) repeated measures analysis of variance (ANOVA) was calculated for FBF and FVC and paired t-tests were used in post hoc analysis. Data are presented as means ± SE throughout and *P* < 0.05 were considered statistically significant.

## Results

### Visit 1: saline versus ascorbic acid in room air

**Figure 2:**
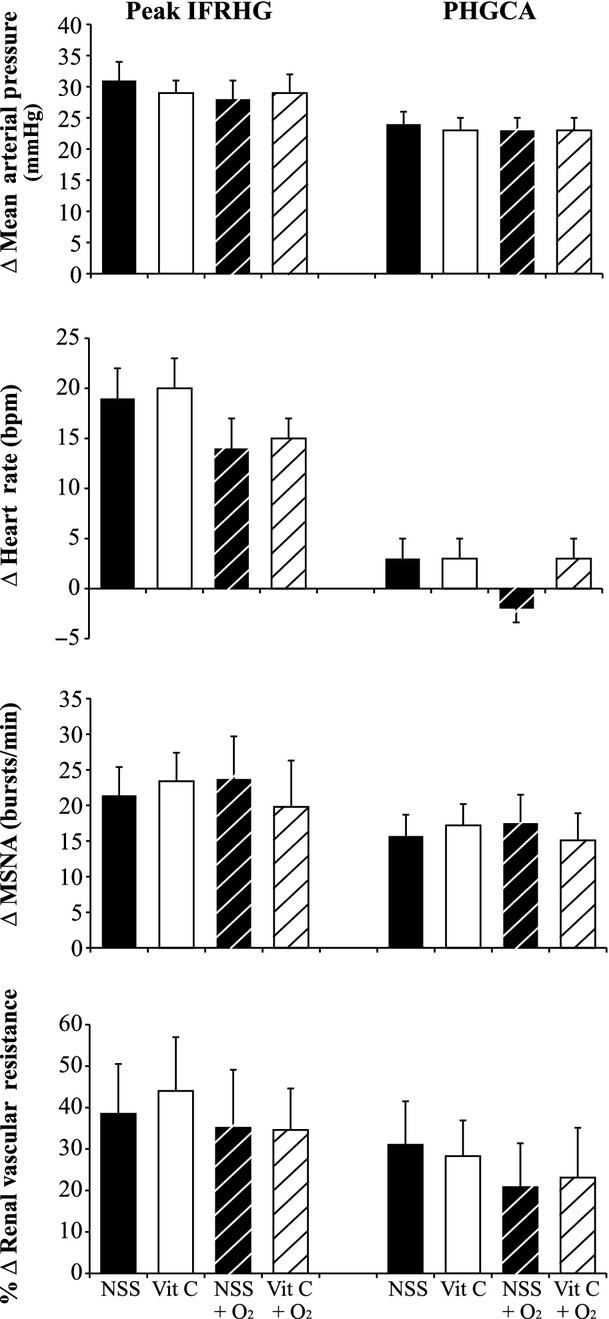
Changes in mean arterial pressure, heart rate, muscle sympathetic nerve activity (MSNA), and renal vascular resistance index in response to ischemic fatiguing rhythmic handgrip (IFRHG) and posthandgrip circulatory arrest (PHGCA). Subjects (*n* = 10) received infusions of normal sterile saline (NSS, black bars) and then ascorbic acid (Vit C, white bars) prior to exercise. On a separate day, subjects (*n* = 9) breathed 100% O_2_ throughout exercise and also received infusions of NSS (black dashed bars) and then Vit C (white dashed bars). Data are M ± SEM.

The subjects had a MVC of 34 ± 3 kg. For the saline trial, they performed IFRHG for 174 ± 14 sec and for the ascorbic acid trial (always performed second) they exercised for 151 ± 15 sec (*P* = 0.030). However, the product of grip force and duration (an index of total work performed) was not different between saline (584 ± 65 kg sec) and ascorbic acid (483 ± 56 kg sec, *P* = 0.184). Neither infusion of saline nor ascorbic acid (range 2551–4856 mg) changed physiological parameters relative to the previous baseline period (Base 1 vs. Base 2, Table 1). As depicted in Figure 2, the reflex increases in MAP (*P* = 0.181), HR (*P* = 0.599), MSNA burst rate (*P* = 0.619), and RVRI (*P* = 0.225) were not different between saline and ascorbic acid. When the muscle metaboreflex was isolated (i.e., PHGCA), ΔMAP (*P* = 0.302), ΔHR (*P* = 0.705), ΔMSNA burst rate (*P* = 0.629), and %ΔRVRI (*P* = 0.782) were not different between infusions.

**Table 1:** Hemodynamic, sympathetic, and renal vascular responses to IFRHG during Visit 1

			Base 1	Base 2	CA	IFRHG first 20	IFRHG peak	PHGCA	Infusion	Time	Interaction
MAP	mm Hg	Saline	84 ± 2	87 ± 3	89 ± 3	94 ± 4	118 ± 4	110 ± 3	0.563	<0.001	0.290
		Ascorbic acid	85 ± 2	88 ± 3	92 ± 3	93 ± 3	117 ± 4	111 ± 4			
HR	bpm	Saline	61 ± 2	62 ± 3	68 ± 2	71 ± 2	81 ± 3	65 ± 3	0.140	<0.001	0.161
		Ascorbic acid	60 ± 2	63 ± 2	69 ± 3	76 ± 3	83 ± 4	66 ± 2			
MSNA	burst/min	Saline	21 ± 3	19 ± 2	16 ± 3	18 ± 3	40 ± 4	35 ± 3	0.793	<0.001	0.928
		Ascorbic acid	19 ± 3	20 ± 4	17 ± 3	19 ± 3	41 ± 4	35 ± 3			
MSNA	total activity	Saline	351 ± 50	325 ± 48	256 ± 55	285 ± 53	1047 ± 134	897 ± 79	0.782	<0.001	0.779
		Ascorbic acid	331 ± 46	321 ± 57	310 ± 61	320 ± 67	1017 ± 123	860 ± 104			
RBV	cm/sec	Saline	50.9 ± 4.2	52.1 ± 3.1	52.3 ± 3.7	54.2 ± 6.9	54.1 ± 7.0	51.7 ± 5.6	0.669	0.895	0.413
		Ascorbic acid	54.9 ± 3.8	54.0 ± 5.0	52.2 ± 4.3	55.4 ± 5.0	52.7 ± 8.7	56.6 ± 5.4			

Subjects (*n* = 10) underwent resting baseline periods before (Base 1) and after (Base 2) infusion of normal sterile saline and then ascorbic acid. Circulatory arrest (CA, inflation of upper arm cuff to suprasystolic pressure) occurred for 1 min prior to the onset of ischemic fatiguing rhythmic handgrip (IFRHG) exercise and the occlusion cuff remained inflated for 2 min of posthandgrip circulatory arrest (PHGCA). Measurements included beat-by-beat mean arterial pressure (MAP), heart rate (HR), muscle sympathetic nerve activity (MSNA), and renal blood flow velocity (RBV). Data are M ± SEM.

### Visit 2: saline versus saline in room air

Six subjects completed a saline–saline time control. For the first trial, they performed IFRHG for 145 ± 8 sec and for the second exercised for 136 ± 15 sec (*P* = 0.613). The product of grip force and duration was not different between the first saline infusion (520 ± 41 kg sec) and second saline infusion (435 ± 63 kg sec, *P* = 0.184). All physiological parameters increased during IFRHG, relative to Base 2 (main effect *P* < 0.001) but there was no order effect. The reflex increases in MAP (29 ± 4 vs. 32 ± 3 mm Hg), HR (18 ± 4 vs. 23 ± 4 bpm), and MSNA burst rate (25 ± 4 vs. 27 ± 5 burst/min) with IFRHG were not different between the first and second infusion. In a similar way, ΔMAP (21 ± 2 vs. 23 ± 1 mm Hg), ΔHR (3 ± 1 vs. 6 ± 4 bpm), and ΔMSNA burst rate (17 ± 3 vs. 20 ± 4 burst/min) were not different between trials during PHGCA.

### Visit 3: saline versus ascorbic acid in hyperoxia

Nine subjects completed Visit 3 and absolute values are presented in Table 2. Relative to the room air saline trial (Visit 1, 175 ± 15 sec), grip duration was similar during the hyperoxia saline trial (152 ± 9 sec, *P* = 0.216). The ΔHR with IFRHG was blunted in eight out of nine subjects with hyperoxia (*P* = 0.071) but ΔMAP (*P* = 0.459), ΔMSNA burst rate (*P* = 0.705), and %ΔRVRI (*P* = 0.468) were similar between room air and hyperoxia (Fig. 2).

**Table 2:** Hemodynamic, sympathetic, renal vascular, and respiratory responses to IFRHG during Visit 3

			Base 1	Base 2	Base 2 + O_2_	CA	IFRHG first 20	IFRHG peak	PHGCA	Infusion	Time	Interaction
MAP	mm Hg	Saline	82 ± 2	86 ± 1	85 ± 2	85 ± 2	88 ± 2	114 ± 3	109 ± 3	0.876	<0.001	0.676
		Ascorbic acid	85 ± 2	86 ± 2	85 ± 2	85 ± 2	86 ± 3	115 ± 4	109 ± 2			
HR	bpm	Saline	59 ± 2	62 ± 3	60 ± 3	63 ± 4	66 ± 4	75 ± 3	60 ± 2	0.660	<0.001	0.292
		Ascorbic acid	59 ± 2	60 ± 3	58 ± 2	62 ± 3	67 ± 4	75 ± 3	62 ± 3			
MSNA	burst/min	Saline	24 ± 5	21 ± 4	20 ± 4	19 ± 4	25 ± 6	49 ± 3	43 ± 2	0.991	<0.001	0.338
		Ascorbic acid	24 ± 5	24 ± 5	22 ± 4	22 ± 3	26 ± 6	44 ± 6	39 ± 3			
MSNA	total activity	Saline	425 ± 71	367 ± 73	365 ± 76	341 ± 80	419 ± 101	1493 ± 199	1234 ± 127	0.880	<0.001	0.991
		Ascorbic acid	429 ± 76	429 ± 70	414 ± 68	345 ± 50	516 ± 163	1470 ± 340	1252 ± 283			
RBV	cm/sec	Saline	46.9 ± 3.6	47.0 ± 1.8	44.3 ± 4.6	47.8 ± 2.7	45.7 ± 6.2	50.8 ± 6.5	52.8 ± 4.3	0.150	0.872	0.634
		Ascorbic acid	49.6 ± 2.2	48.0 ± 3.4	48.3 ± 3.4	51.4 ± 5.3	50.2 ± 5.5	48.6 ± 4.9	51.3 ± 6.0			
SpO_2_	%	Saline	–	97.6 ± 0.2	99.4 ± 0.3	–	–	99.6 ± 0.3	99.6 ± 0.3	0.987	<0.001	0.577
		Ascorbic acid	–	97.6 ± 0.3	99.5 ± 0.2	–	–	99.4 ± 0.3	99.7 ± 0.3			
EtCO_2_	mm Hg	Saline	–	43 ± 2	43 ± 2	–	–	42 ± 2	38 ± 2	0.134	<0.001	0.543
		Ascorbic acid	–	42 ± 2	41 ± 2	–	–	40 ± 2	36 ± 2			
MV	L/min	Saline	–	8.1 ± 1.0	8.9 ± 1.0	–	–	10.7 ± 1.3	11.6 ± 2.1	0.491	0.030	0.844
		Ascorbic acid	–	8.5 ± 0.3	9.2 ± 0.8	–	–	12.1 ± 2.1	11.8 ± 2.0			

Subjects (*n* = 9) underwent resting baseline periods before (Base 1) and after (Base 2) infusion of normal sterile saline and then ascorbic acid. Circulatory arrest (CA, inflation of upper arm cuff to suprasystolic pressure) occurred for 1 min prior to the onset of ischemic fatiguing rhythmic handgrip (IFRHG) exercise and the occlusion cuff remained inflated for 2 min of posthandgrip circulatory arrest (PHGCA). Measurements included beat-by-beat mean arterial pressure (MAP), heart rate (HR), muscle sympathetic nerve activity (MSNA), renal blood flow velocity (RBV), arterial oxygen saturation (SpO_2_), end tidal carbon dioxide (EtCO_2_), and minute ventilation (MV). Data are M ± SEM.

Relative to the hyperoxia saline trial, the hyperoxia ascorbic acid trial (always performed second) resulted in shorter grip durations (132 ± 9 sec, *P* = 0.004) and the product of grip force and duration was also lower with ascorbic acid (478 ± 84 vs. 394 ± 76, *P* = 0.005). Moreover, exposure to 100% oxygen increased oxygen saturation and the IFRHG protocol increased minute ventilation (Table 2) but this was not different between infusions. During PHGCA, the subjects developed modest hypocapnia. As shown in Figure 2, the reflex changes in MAP (*P* = 0.811), HR (*P* = 0.411), MSNA burst rate (*P* = 0.104), and RVRI (*P* = 0.957) were not significantly different between hyperoxia saline and hyperoxia ascorbic acid.

### Follow-up study

**Figure 3:**
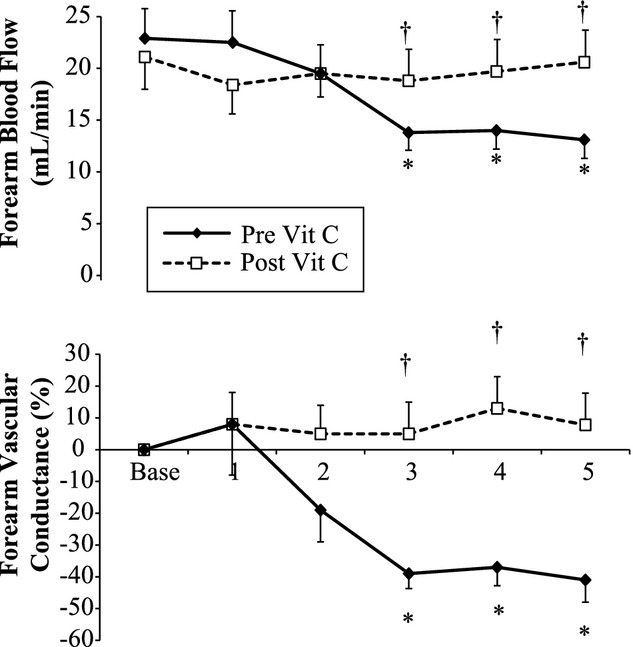
Forearm blood flow and forearm vascular conductance during exposure to 5 min of 100% oxygen at rest (*n* = 8). In this follow-up study, the first trial occurred before infusion of ascorbic acid (Pre Vit C) and the second trial occurred after infusion of ascorbic acid (Post Vit C). During the baseline period, subjects breathed room air. *Indicates a significant reduction from the preceding baseline, †indicates a significant difference between trials. Data are M ± SEM.

Exposure to 100% oxygen raised arterial oxygen saturation in all trials (from 98 ± 1 to 99 ± 1, *P* < 0.001). Relative to the room air baseline period, exposure to 100% hyperoxia reduced FBF and FVC under control conditions (no infusion, Fig. 3 solid line). Specifically, at minute 3 (*P* = 0.004), minute 4 (*P* = 0.008), and minute 5 (*P* = 0.010) of hyperoxia under control conditions, FBF and FVR were reduced compared to the preceding room air baseline. During the infusion of ascorbic acid, the forearm vasoconstriction effect was abolished (Fig. 3, dashed line). At minute 3 (*P* = 0.030), minute 4 (*P* = 0.040), and minute 5 (*P* = 0.040), FBF was significantly greater under ascorbic acid infusion compared with control. The changes in HR (control: from 59 ± 2 to 57 ± 3 bpm; ascorbic acid from 59 ± 2 to 57 ± 2 bpm) and MAP (control: from 84 ± 1 to 85 ± 2 mm Hg; ascorbic acid from 84 ± 2 to 86 ± 1 mm Hg) in response to hyperoxia were similar under both conditions.

Under control conditions, venous blood levels of ascorbic acid were similar before hyperoxia (0.89 ± 0.07 mg/dL) and at the end of the fifth minute of hyperoxia (0.90 ± 0.08 mg/dL). Following the initial 100 mL of ascorbic acid (immediately before the second hyperoxia trial), plasma levels of ascorbic acid were 13.2 ± 1.4 mg/dL and at the end of the fifth minute of hyperoxia plasma levels were 13.3 ± 1.9 mg/dL.

## Discussion

The purpose of this study was to determine the effect of oxidative stress on the EPR in healthy young humans. By using ascorbic acid infusion (Visit 1) and brief hyperoxia inhalation (Visit 3), we tested whether ROS impact the reflex cardiovascular adjustments to IFRHG. Herein, we demonstrate that neither ascorbic acid infusion nor hyperoxia inhalation affect the EPR in healthy young humans. While contrary to our initial hypotheses, these findings suggest that ROS do not play a significant role in the normal reflex adjustments to acute ischemic exercise.

Skeletal muscle contraction, particularly under hypoperfused conditions, generates an accumulation of potassium (Rybicki et al. 1984), lactate (Sinoway et al. 1993), bradykinin (Stebbins and Longhurst 1986), arachidonic acid metabolites (Rotto et al. 1990), ATP (Li et al. 2008), and diprotonated phosphate (Sinoway et al. 1994) within the working muscle. Both human and animal experiments have indicated that these metabolites can stimulate group III and group IV muscle afferents, ultimately leading to increased sympathetic outflow, tachycardia, hypertension, and hyperpnea (McCloskey and Mitchell 1972). The concept that ROS are capable of stimulating muscle afferents was first demonstrated by Delliaux et al. (Delliaux et al. 2009) using a rat model. Specifically, these investigators showed that intramuscular injection of hydrogen peroxide activated group IV afferents and this effect could be prevented by superoxide dismutase (SOD). Importantly, group IV activity in response to rhythmic contractions of the tibialis anterior was also attenuated by SOD in this study (Delliaux et al. 2009). Wang et al. (Wang et al. 2009) documented that both tempol (a membrane permeable SOD mimetic) and apocynin (decreases NADPH oxidase-derived ROS) attenuated the EPR in response to a 30-second static contraction in healthy rats. These findings, although not universally accepted (Bonigut et al. 1996; Koba et al. 2009; McCord et al. 2011), suggested that ROS might sensitize muscle afferents and play an excitatory role in the EPR in healthy animals. Translating previous animal experiments into a human model was the impetus for the current investigation.

During Visit 1, subjects received an intravenous infusion of high dose ascorbic acid prior to and during the exercise paradigm. Our laboratory recently demonstrated that this dose of ascorbic acid attenuated the EPR in patients with PAD (Muller et al. 2012). Other studies in healthy people have indicated that systemic infusion of ascorbic acid (ranging from 3000–5000 mg) does not alter resting MAP, HR, or MSNA but significantly lowers plasma markers of oxidative stress (Bell et al. 2003; Monahan et al. 2004; Bruno et al. 2012). The current study was not focused on plasma ROS but rather ROS within skeletal muscle that are thought to stimulate muscle afferents. Because recording from muscle afferents is not technically possible in humans, we chose a powerful stimulus (i.e., fatiguing small muscle exercise under ischemic conditions) that would have allowed us to detect physiological changes in HR, MAP, MSNA, and RVRI had ROS indeed been involved in the EPR. Contrary to our hypothesis, ascorbic acid did not alter ΔHR, ΔMAP, ΔMSNA, or ΔRVRI in responses to IFRHG or PHGCA compared with saline (Fig. 2). These data indicate that ROS do not play a significant role in the EPR in healthy humans. Regardless of the index of sympathoexcitation (MSNA or RVRI) and regardless of the stimulus (IFRHG or PHGCA), our findings suggest that the EPR is not reduced by ascorbic acid. Time control experiments (Visit 2) give additional evidence that the IFRHG and PHGCA paradigm used in the current study acutely elevated HR, MAP, MSNA, and RVRI; these data are consistent with previous human studies (Mark et al. 1985; Victor et al. 1987; Cui et al. 2010, 2011; Delaney et al. 2010).

Hyperoxia inhalation inhibits the carotid chemoreflex (Nye et al. 1981) and is a potent vasoconstrictor (Jamieson 1989; Mak et al. 2002; Rousseau et al. 2005) because it produces high amounts of superoxide anions that subsequently reduce nitric oxide bioavailability (Gryglewski et al. 1986; Rubanyi and Vanhoutte 1986; Zhilyaev et al. 2003; McNulty et al. 2005). Inhibition of prostaglandin synthesis by hyperoxia may also play a role in the vasoconstrictor response (Rousseau et al. 2010). To further challenge our hypothesis that ROS play a role in the EPR, subjects breathed 100% oxygen during Visit 3. Contrary to our original hypothesis, this brief period of hyperoxia did not augment the EPR (comparing saline Visit 1 to saline Visit 3) and it also did not affect resting MAP, HR, or MSNA. Two previous studies have measured MSNA during exercise combined with hyperoxia and warrant brief discussion. Seals et al. (1991) first showed that 3–4 min of hyperoxia lowered MSNA at rest but did not affect ΔHR, ΔMAP, or ΔMSNA during 3–4 min of rhythmic handgrip at 50% MVC. On the other hand, Houssiere et al. (Houssiere et al. 2006) documented that 15 min of hyperoxia lowered MAP and MSNA at rest; these investigators found that ΔMAP and ΔMSNA were augmented with hyperoxia during static handgrip at 30% MVC as well as during a subsequent period of PHGCA. The lack of consistency in these studies (Seals et al. 1991; Houssiere et al. 2006) may be attributed to different exercise interventions and/or different durations of hyperoxia than the current study. Importantly, these cited studies did not intend to study oxidative stress mechanisms but rather the interactions between the carotid chemoreflex and the EPR.

Part of this study (Visit 3) included ascorbic acid infusion combined with hyperoxia. While there were no significant differences between saline and ascorbic acid regarding the EPR with hyperoxia during Visit 3, recent work from our laboratory has shown that infusion of 3.0 grams of ascorbic acid can prevent the impairment in coronary blood flow and myocardial function caused by hyperoxia (McNulty et al. 2007; Gao et al. 2012). Our follow-up study measuring FBF and FVC in response to hyperoxia and ascorbic acid confirms and extends upon these prior studies. These cited studies showing the effectiveness of intravenous ascorbic acid on vascular responses stimulated our original hypothesis that antioxidant infusion would attenuate the augmented EPR (i.e., a neural reflex) caused by hyperoxia. Because the EPR was not augmented by hyperoxia in our study, ascorbic acid was not able to attenuate a response. It should be emphasized that data collection had to be completed prior to analyzing the physiological responses (i.e., the ascorbic acid was infused during Visit 3 prior to us finding that the EPR was not augmented by hyperoxia). Our findings are consistent with a recent study showing that alpha-adrenergic vasoconstriction did not restrict exercise blood flow to a greater extent under hyperoxic conditions (Casey et al. 2013). Taken together, hyperoxia is capable of acutely reducing FVC through an oxidative stress mechanism but our exercise data indicate it does not affect sympathetically mediated vasoconstriction to inactive skeletal muscle or the renal circulation. The interaction between ROS-mediated vasoconstriction and neurally mediated vasoconstriction is an area for future investigation.

While MSNA is a direct measure of sympathetic nerve traffic, acute changes in RVRI can also indicate sympathoexcitation. The renal vasculature receives ∼20% of cardiac output at rest and is innervated by sympathetic nerves. In response to exercise or orthostatic stress, alpha-adrenergic mediated renal vasoconstriction occurs in an effort to redistribute blood flow to critical organs (Momen et al. 2003, 2005; Sauder et al. 2008; Conboy et al. 2010). To our knowledge, the current data are the first report of RBV and RVRI in response to IFRHG. IFRHG is considered to be a similar yet distinct stimulus compared to rhythmic handgrip exercise under freely perfused conditions (Cook and Ray 2009). Herein, we demonstrated an ∼40% increase in RVRI at peak exercise and a 25–35% increase in RVRI during PHGCA, relative to pre-exercise baseline. These findings are consistent with previous studies from our laboratory (Momen et al. 2003, 2008) and others (Kuipers et al. 2009) characterizing acute changes in renal vascular tone in response to isometric handgrip exercise. In the current study, RBV was relatively stable (Table 1 and 2) but, in the face of an increased MAP, RVRI was significantly elevated therefore indicating renal vasoconstriction. Our data support the concept that sympathetic outflow to the kidney is increased during IFRHG and PHGCA but is not significantly affected by interventions that acutely increase oxidative stress in young healthy humans.

### Experimental considerations

The current study used small muscle ischemic exercise to isolate reflex neurovascular changes in response to IFRHG and PHGCA that might be altered by skeletal muscle ROS. However, it is possible that larger muscle exercise under freely perfused conditions may elicit a different response. Plasma blood markers of ROS, commonly obtained in whole-body exercise studies (Rietjens et al. 2007), do not necessarily reflect ROS reaching the sensory muscle afferents so we chose not to quantify plasma ROS. The lack of relationship between oxidative stress blood markers and afferent stimulation may be especially true under ischemic exercise conditions where lactate ions and/or acidosis may inhibit (Groussard et al. 2000) or potentiate (Siesjo et al. 1985) skeletal muscle ROS production. Previous experiments from our laboratory (Sinoway et al. 1992; McClain et al. 1993) have shown that muscle pH is reduced to ∼6.3–6.7 during handgrip, which may either increase or decrease ROS production within the muscle. There is also likely to be considerable redundancy between these metabolites, skeletal muscle afferents, and sympathetic outflow. Thus, it is possible that none of our interventions truly altered oxidative stress at the level of the muscle afferent and future studies using new approaches may be able to test this more directly. Additionally, ascorbic acid has direct vascular effects and it recycles tetrahydrobiopterin, a necessary cofactor for endothelial nitric oxide synthase (Traber and Stevens 2011). Nevertheless, our rigorously controlled experiments provided maximal antioxidant (high dose ascorbic acid) and prooxidant (100% hyperoxia along with IFRHG) stimuli to the human body. As such, we are confident that if ROS played a significant role in the EPR we would have been able to detect it with this experimental design.

It is also important to emphasize that the current studies were acute in nature and it is possible that long-term exposure to ROS (e.g., cigarette smokers or patients receiving supplemental oxygen) may induce phenotypic changes in muscle afferents. In a similar way, longer exposure to ascorbic acid may be needed to affect the cardiovascular responses to ischemic exercise.

Surprisingly, in Visits 1 and 3, we found that grip duration was shorter during ascorbic acid compared with saline. We originally attributed this to residual fatigue as ascorbic acid infusion always occurred second (Seals and Enoka 1989). However, this effect was not seen during Visit 2 when saline was given twice. This suggests that ascorbic acid itself may impair muscle contractile performance. While currently an area of active research (Fisher-Wellman et al. 2009; Nikolaidis et al. 2012), we are unaware of studies evaluating the effect of ascorbic acid on ischemic exercise performance and its physiological relevance is yet to be determined.

## Conclusion

Elevated oxidative stress and an augmented EPR are both hallmarks of many cardiovascular diseases but the interaction between ROS and the sympathetic nervous system is not clear. Animal experiments have shown that ROS can stimulate a variety of afferents (Huang et al. 1995; Li et al. 1996; Delliaux et al. 2009), thereby initiating sympathetic reflexes. The current data are the first to experimentally isolate the interaction between ROS and the EPR in healthy humans. Specifically, we have shown that neither ascorbic acid infusion nor brief hyperoxia inhalation affect the ΔHR, ΔMAP, ΔMSNA, or ΔRVRI in response to ischemic exercise, compared with control conditions. ROS levels are acutely elevated by muscle contraction (Karamouzis et al. 2004; Rietjens et al. 2007) and are chronically elevated in hypertension, heart failure, and PAD (Schnabel and Blankenberg 2007). Future studies relating to ROS and sympathoexcitation during exercise in these patient groups are much needed.
